# AIPFusion-Net: an interaction-fusion framework for anti-inflammatory peptide prediction using protein language model embeddings, local sequence patterns, and handcrafted descriptors

**DOI:** 10.3389/fmolb.2026.1952395

**Published:** 2026-10-05

**Authors:** Wentao Wang, Weidong Yang, Hongmei Li, Zhenyu Cui, Dan Shen

**Affiliations:** 1 Department of Urology, Affiliated Hospital of Hebei University, Baoding, Hebei, China; 2 Department of Emergency, Affiliated Hospital of Hebei University, Baoding, Hebei, China

**Keywords:** AIPFusion-Net, anti-inflammatory peptides, interaction fusion, protein language models, sequence motifs

## Abstract

**Introduction:**

Anti-inflammatory peptides (AIPs) are promising candidates for treating inflammatory diseases. However, experimental identification is time-consuming and costly, creating a need for accurate computational prediction methods.

**Methods:**

We developed AIPFusion-Net, an interaction-fusion framework that integrates three complementary sources of peptide information: global contextual embeddings generated by the ESM2 protein language model, local sequence patterns extracted from biologically relevant subsequences, and handcrafted physicochemical and sequence descriptors. These representations were integrated through an interaction-fusion module and evaluated on two independent test datasets, Ind-1049 and Ind-426.

**Results:**

On the Ind-1049 dataset, AIPFusion-Net achieved an accuracy of 84.6%, sensitivity of 71.7%, specificity of 89.3%, MCC of 0.654, and AUC of 0.884. On the Ind-426 dataset, it achieved an accuracy of 82.3%, sensitivity of 84.4%, specificity of 76.9%, MCC of 0.673, and AUC of 0.882. The model outperformed the comparative predictors in overall classification performance and discriminative ability.

**Discussion:**

The results indicate that combining global contextual information, local sequence patterns, and handcrafted descriptors through interaction fusion improves the representation and identification of AIPs. AIPFusion-Net provides a useful computational framework for AIP screening and may support the discovery and design of novel anti-inflammatory peptides.

## 1 Introduction

Anti-inflammatory peptides (AIPs) are a class of bioactive peptides that play an essential role in regulating immune responses and alleviating inflammation (Medzhitov, 2010; Ferrero-Miliani et al., 2007). These peptides have gained significant attention as potential therapeutic agents for treating chronic inflammatory diseases, autoimmune disorders, and infections. Identifying AIPs from peptide sequences is a challenging task due to their structural diversity and the subtle sequence variations that determine their biological activity (Deepak et al., 2019; Majumder et al., 2016). Experimental identification methods, while effective, are resource-intensive, time-consuming, and often lack the high-throughput capacity needed for large-scale studies (Bian et al., 2021). Consequently, there is a growing demand for computational tools that can accurately predict AIPs based on sequence information (Xiao et al., 2025).

In recent years, machine learning (ML) and deep learning (DL) techniques have shown great promise in peptide prediction, leveraging vast amounts of sequence data to uncover hidden patterns and relationships. For example, Khatun et al. (2020) introduced ProIn-Fuse, a model integrating multiple sequence-based feature representations and probabilistic scores, achieving superior performance over existing predictors. Manavalan et al. (2018) proposed AIPpred, a random forest–based predictor utilizing optimized sequence-derived features, which demonstrated robust predictive performance on independent datasets. To facilitate peptide-based therapeutic development, Alotaibi et al. (2021) presented AntiFlamPred, a deep learning–based predictor combining encoded features with rigorous validation, achieving high accuracy and stability. Similarly, Zhao et al. (2021) developed iAIPs, a random forest–based model with optimized sequence-derived features, providing a foundation for evolutionary analysis of peptides. Zhang et al. (2021) proposed a predictor for anti-inflammatory epitopes (AIEs) that integrates multiple sequence features via an ensemble classifier chain, achieving high accuracy in both cross-validation and independent tests. Guan et al. (2023) introduced an ensemble framework combining extremely randomized trees, GRU, and CNN with attention, yielding superior performance and insights for therapeutic design. Khatun et al. (2019) also presented PreAIP, a random forest predictor integrating sequence, evolutionary, and structural features, outperforming previous methods. Additional contributions include AIPStack by Deng et al. (2022), a two-layer stacking ensemble model using hybrid sequence features to highlight key sequence determinants, and AIPs-SnTCN by Raza et al. (2023), which combines word embeddings, conjoint triad features, and a self-normalized temporal convolutional network, achieving high accuracy and AUC. Gaffar et al. (2024) proposed AIPs, a voting classifier integrating eight feature descriptors and five machine-learning models, demonstrating superior accuracy and MCC across independent datasets. Xu et al. (2024) introduced DAC-AIPs, a deep learning model combining variational autoencoder and contrastive learning with multi-hot encoding, offering enhanced accuracy and interpretability. Zhu et al. (2024) developed DeepAIP, a contextual self-attention deep learning model using Prot-T5 features, successfully identifying novel peptides with high accuracy and MCC.Finally, Yan et al. (2022) proposed TPpred-ATMV, an adaptive multi-view tensor learning model integrating diverse sequence features to predict multiple types of therapeutic peptides, while Guo et al. (2021) developed PreTP-EL, an ensemble learning–based predictor that fuses multiple feature types, achieving superior performance over existing methods.Emerging models such as PepNet (Han et al., 2024) and PreTP-Stack (Yan et al., 2023) further illustrate the trend of integrating deep learning and ensemble strategies to predict a wide range of therapeutic peptides with improved efficiency and reliability. Collectively, these computational methods demonstrate the growing potential of ML and DL in accelerating peptide-based therapeutic discovery.

However, most existing computational models for AIP prediction rely on either global sequence features or handcrafted descriptors, which typically fail to capture the full complexity of peptide structures and interactions. Furthermore, the majority of these methods do not fully account for the intricate local sequence motifs and contextual information that may be critical for determining the biological activity of AIPs. As a result, there is a need for a more holistic approach that integrates multiple levels of sequence information and captures both local and global sequence patterns.

To address these challenges, we propose AIPFusion-Net, a task-specific interaction-fusion framework designed for anti-inflammatory peptide prediction (Figure 1). Unlike conventional feature fusion strategies that simply combine heterogeneous representations, AIPFusion-Net aims to explicitly model the interactions among multi-scale peptide representations to better capture the discriminative characteristics of short AIP sequences. Specifically, AIPFusion-Net integrates three complementary sources of information, including protein language model embeddings, biologically guided local sequence patterns, and handcrafted physicochemical descriptors. The protein language model embeddings derived from ESM-2 capture global contextual semantic information within peptide sequences, while the local sequence branch employs biologically meaningful sequence windows to identify localized motifs and functional patterns associated with anti-inflammatory activity. In addition, handcrafted descriptors provide complementary physicochemical and sequence-level information, including amino acid composition, dipeptide composition, and other properties related to peptide functionality. Rather than directly concatenating these heterogeneous representations, AIPFusion-Net introduces an interaction-fusion module to explicitly learn the complementary relationships among global, local, and physicochemical features. This interaction-aware strategy enables the model to generate more discriminative multi-scale peptide representations and is particularly suitable for short AIP sequences, where functional determinants are often sparse and locally distributed. To evaluate the performance of the proposed model, we tested AIPFusion-Net on two independent benchmark datasets, Ind-1049 and Ind-426, containing diverse AIPs and non-AIPs. The results demonstrate that AIPFusion-Net achieves superior performance compared with existing state-of-the-art methods across multiple evaluation metrics, including accuracy, sensitivity, specificity, Matthews correlation coefficient (MCC), and area under the receiver operating characteristic curve (AUC).

**FIGURE 1:**
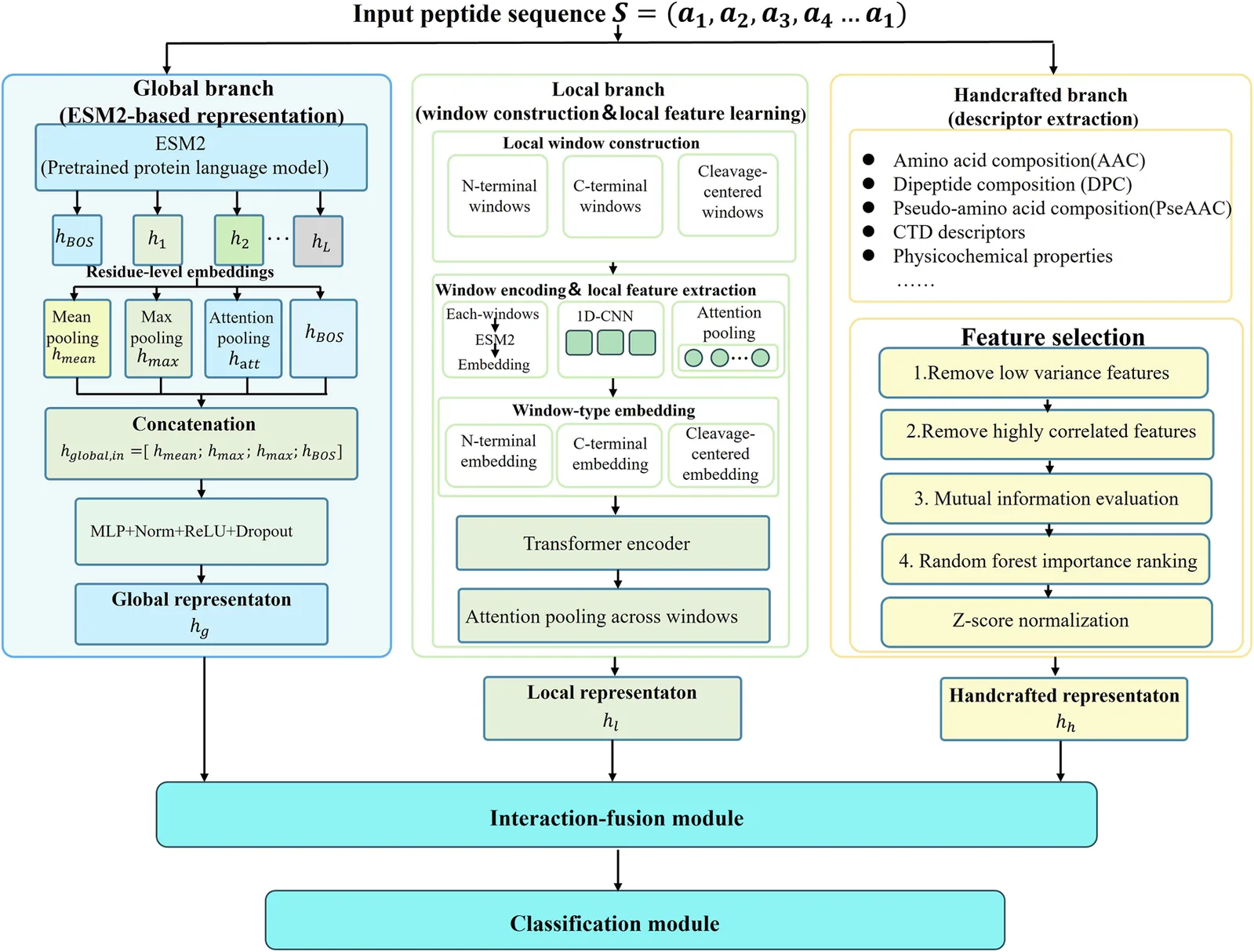
Schematic overview of the proposed AIPFusion-Net framework for anti-inflammatory peptide prediction.

The primary contributions of this work are as follows:

1. We propose AIPFusion-Net, a task-specific interaction-aware fusion framework that integrates global contextual representations, local sequence patterns, and physicochemical descriptors for anti-inflammatory peptide prediction.
2. We demonstrate the effectiveness of the proposed model through extensive experiments on two benchmark datasets (Ind-1049 and Ind-426), where it outperforms existing AIP prediction methods.
3. We provide a deeper understanding of the key sequence features that contribute to AIP activity, which could be valuable for future research and the design of novel anti-inflammatory peptides.
4. We propose an interaction-fusion strategy that effectively combines complementary sequence representations, enhancing the model’s ability to capture both the broad contextual information and the fine-grained local patterns that are crucial for AIP prediction.

## 2 Materials and methods

### 2.1 Overview of AIPFusion-Net

In this study, we developed AIPFusion-Net, an interaction-fusion framework for the prediction of anti-inflammatory peptides (AIPs). The proposed model was designed to integrate complementary sequence information from multiple representational levels in order to improve the discrimination between AIPs and non-AIPs. Specifically, AIPFusion-Net combines global contextual embeddings derived from a pretrained protein language model, local motif-aware representations extracted from biologically relevant subsequences, and handcrafted descriptors reflecting compositional, physicochemical, and biologically inspired sequence characteristics. Given an input peptide sequence $S=\left(a_{1},a_{2},\ldots ,a_{L}\right)$ , AIPFusion-Net learns a global representation $h_{g}$ , a local representation $h_{l}$ , and a handcrafted feature representation $h_{h}$ , which are subsequently integrated through an interaction-fusion module for final prediction. The overall framework can be formulated as shown in Equation 1:

$$\hat{y}=\sigma \left(f_{\text{cls}}\left(f_{\text{fusion}}\left(h_{g},h_{l},h_{h}\right)\right)\right)$$ (1)

where $\hat{y}$ denotes the predicted probability that the input peptide is anti-inflammatory, $f_{\text{fusion}}\left(\cdot \right)$ denotes the feature fusion function, $f_{\text{cls}}\left(\cdot \right)$ represents the classification module, and $\sigma \left(\cdot \right)$ is the sigmoid activation function.

### 2.2 Data set

The quality of the training data plays a critical role in developing a robust and generalizable deep-learning model. In this study, the benchmark dataset was constructed based on the dataset reported in the IF-AIP study (Gaffar et al., 2024). The original dataset contained 1,962 anti-inflammatory peptides (AIPs) and 2,896 non-AIPs. To reduce sequence redundancy, CD-HIT (Huang et al., 2010) was applied with a sequence identity threshold of 0.9. No overlapping peptide sequences were retained between the training dataset and the independent test datasets, ensuring that the independent evaluation was performed on unseen samples.After removing redundant sequences, the final training dataset included 3,790 samples, consisting of 1,451 AIPs and 2,339 non-AIPs.

To evaluate the generalization ability of the proposed model, two independent test datasets, namely, Ind-1049 (Zhao et al., 2021) and Ind-426 (Gupta et al., 2017), were further employed. The Ind-1049 dataset contains 420 AIPs and 629 non-AIPs, while the Ind-426 dataset includes 173 AIPs and 253 non-AIPs. To reduce homology bias, sequence identity thresholds of 0.8 and 0.6 were used for Ind-1049 and Ind-426, respectively. In addition, no overlapping sequences were retained between the training set and the independent test sets, ensuring an unbiased evaluation of model performance.

The detailed class distributions of the training dataset and the two independent test datasets are summarized in Table 1.

**TABLE 1:** Class distribution of the training and independent test datasets for anti-inflammatory peptide prediction.

Data set	AIPs	Non-AIPs
Training	1451	2339
Ind-1049	420	629
Ind-426	173	253

### 2.3 Protein language model-based representation

To capture deep contextual information from peptide sequences, the pretrained protein language model ESM2 (Lin et al., 2023) was employed as the backbone encoder. Specifically, ESM2 (esm2_t33_650M_UR50D) was used to generate residue-level contextual representations, with a hidden embedding dimension of 1280. During model training, the parameters of the pretrained ESM2 encoder were kept frozen and only used as a feature extractor to obtain sequence representations.For a given peptide sequence S , ESM2 generates residue-level embeddings $H=\left[h_{1},h_{2},\ldots ,h_{L}\right]$ , where $h_{i}\in R^{d}$ , together with a beginning-of-sequence embedding $h_{\text{BOS}}$ . Based on these outputs, several sequence-level summaries were constructed to characterize the peptide from complementary perspectives. The mean-pooled representation was defined as shown in Equation 2:

$$h_{\text{mean}}=\frac{1}{L}\sum_{i=1}^{L}h_{i}$$ (2)

The max-pooled representation $h_{\max}$ was obtained by taking the maximum value across all residue embeddings in each dimension. To further emphasize informative residues, an attention-pooling mechanism was introduced (Rao et al., 2021). The attention weight of the i -th residue was computed as shown in Equation 3:

$$\alpha_{i}=\frac{\exp\left(e_{i}\right)}{\sum_{k=1}^{L}\exp\left(e_{k}\right)}$$ (3)

where $e_{i}$ denotes the attention score of the i -th residue. The attention-pooled representation was then defined in Equation 4:

$$h_{att}=\sum_{i=1}^{L}\alpha_{i}h_{i}$$ (4)

These ESM2-derived features were concatenated as shown in Equation 5.

$$h_{{\text{global}}_{\text{in}}}=\left[h_{\text{BOS}};h_{\text{mean}};h_{\max};h_{\text{att}}\right]$$ (5)

This representation enables the model to capture the overall semantic and contextual information of the peptide sequence.

### 2.4 Local window construction and local feature learning

Although full-sequence embeddings provide a strong global characterization of peptides, biologically relevant signals are often concentrated in local subsequences. To capture such local information, multiple subsequence windows were constructed for each peptide, including N-terminal windows, C-terminal windows, and cleavage-centered windows. For terminal regions, sequence windows with lengths of 10 and 20 residues were extracted from both the N-terminus and C-terminus.Terminal windows were extracted directly from the sequence ends using predefined lengths, whereas cleavage-centered windows were generated around candidate cleavage-related positions identified from basic residues and short motifs, including K, R, KR, RR, RK, and KK. Specifically, individual K and R residues were considered as candidate cleavage-associated positions. For adjacent basic motifs, including KR, RR, RK, and KK, the corresponding motif centers were additionally identified as candidate positions. Duplicate positions generated from overlapping residues or motifs were removed before window extraction. For each peptide, a maximum of six cleavage-centered windows were retained. For a cleavage position p , the corresponding local window was defined as Equation 6:

$$S_{p}^{\text{clv}}=\left(a_{\max\left(1,p-r\right)},\ldots ,a_{p},\ldots ,a_{\min\left(L,p+r\right)}\right)$$ (6)

where r denotes the predefined window radius.

Each local window was independently encoded by ESM2 to generate residue-level contextual embeddings. These embeddings were subsequently processed by a one-dimensional convolutional neural network with multiple kernel sizes (kernel sizes of 3 and 5), allowing the model to capture local sequence patterns at different receptive fields. The resulting residue-level features were then aggregated by attention pooling to generate a fixed-length representation for each window. To preserve the biological origin of each window, a learnable window-type embedding was introduced to indicate whether the corresponding subsequence originated from the N-terminus, C-terminus, or a cleavage-centered region. The sequence of type-aware window embeddings was further modeled by a Transformer encoder, which enabled the model to capture dependencies and interactions among multiple local regions within the same peptide. Finally, attention pooling (Vaswani et al., 2017) was applied across all windows to obtain the final local representation $h_{l}$ . Through this design, the local branch was able to capture both individual motif-like patterns and the contextual relationships among distinct functional subsequences.

### 2.5 Handcrafted feature extraction

In addition to deep contextual representations, handcrafted sequence descriptors were extracted to provide complementary compositional and physicochemical information. These descriptors included amino acid composition, dipeptide composition, pseudo-amino acid composition, composition-transition-distribution descriptors, physicochemical properties, and biologically motivated sequence features (Chou, 2001; Dubchak et al., 1999; Chen et al., 2018). For a peptide of length L , the amino acid composition of residue type i was defined as Equation 7:

$$AAC_{i}=\frac{n_{i}}{L}$$ (7)

where $n_{i}$ denotes the occurrence count of the i -th amino acid in the sequence. Similarly, the dipeptide composition was defined as Equation 8:

$$DPC_{i}=\frac{d_{i}}{L-1}$$ (8)

where $d_{i}$ denotes the occurrence count of the corresponding adjacent amino acid pair.

Pseudo-amino acid composition was employed to incorporate both residue composition and short-range sequence-order information, whereas CTD descriptors were used to characterize the global distribution of grouped physicochemical properties, such as hydrophobicity, polarity, charge, polarizability, solvent accessibility, and secondary structure propensity.

In addition, a series of physicochemical descriptors was calculated, including peptide length, molecular weight, isoelectric point, net charge, hydrophobicity-related properties, aromaticity, and aliphatic index. The net charge at a given pH was estimated according to Equation 9:

$$Q\left(\text{pH}\right)=\sum_{r\in \text{basic}}\frac{1}{1+10^{\left(\text{pH}-pK_{a}\left(r\right)\right)}}-\sum_{r\in \text{acidic}}\frac{1}{1+10^{\left(pK_{a}\left(r\right)-\text{pH}\right)}}$$ (9)

To further characterize sequence patterns potentially associated with peptide processing and anti-inflammatory activity, several biologically motivated descriptors were also included, such as basic residue frequency, cleavage-related motif density, terminal residue patterns, and terminal hydrophobicity. All handcrafted descriptors were concatenated into a unified feature vector and used as the input to the handcrafted branch.

### 2.6 Handcrafted feature selection

As the handcrafted feature space was high-dimensional and potentially redundant, a multi-step feature selection procedure was performed on the training set. Initially, 626 handcrafted descriptors were generated, including amino acid composition, dipeptide composition, CTD descriptors, pseudo-amino acid composition, physicochemical properties, and biological sequence features. First, features with extremely low variance were removed. Second, highly correlated features were filtered out to reduce redundancy (Peng et al., 2005; Breiman, 2001; Guyon et al., 2002). Subsequently, mutual information was used to evaluate the relevance of each feature with respect to the class label, which can be expressed as Equation 10:

$$I\left(X;Y\right)=\sum_{x\in X}\sum_{y\in Y}p\left(x,y\right)\log\frac{p\left(x,y\right)}{p\left(x\right)p\left(y\right)}$$ (10)

Finally, a random forest model was employed to rank the remaining features according to their relative importance, and the most informative subset was retained. After the selection procedure, the top 80 features were retained as the final handcrafted representation for model training. After feature selection, z-score normalization was applied to standardize the handcrafted features Equation 11:

$$x^{\prime }=\frac{x-\mu }{\sigma }$$ (11)

where μ and σ denote the mean and standard deviation estimated from the training set, respectively. The same transformation was subsequently applied to the independent test set.

### 2.7 Model architecture and interaction fusion

The global branch takes $h_{\text{global}\_\text{in}}$ as input and projects it into a compact latent representation $h_{g}$ through a multilayer perceptron composed of linear transformation, normalization, nonlinear activation, and dropout. The local branch generates the representation $h_{l}$ by integrating local convolution, window-type encoding, Transformer-based interaction modeling, and attention aggregation. The handcrafted branch maps the selected descriptor vector into a latent representation $h_{h}$ through nonlinear projection and residual learning.

To integrate these three complementary representations, AIPFusion-Net adopts an interaction-fusion strategy. In addition to the original branch outputs, pairwise interaction features are constructed using element-wise multiplication and absolute difference operations. The fused representation is defined as Equation 12:

$$h_{f}=\left[h_{g};h_{l};h_{h};h_{g}⊙h_{l};h_{g}⊙h_{h};h_{l}⊙h_{h};|h_{g}-h_{l}|;|h_{g}-h_{h}|;|h_{l}-h_{h}|\right]$$ (12)

where ⊙ denotes element-wise multiplication. This formulation enables the model to explicitly capture the consistency, complementarity, and discrepancy among different representation sources. The fused vector is then fed into a multilayer classifier to obtain the final prediction probability (Equation 13):

$$\hat{y}=\sigma \left(f_{\text{cls}}\left(h_{f}\right)\right)$$ (13)

where $\sigma \left(\cdot \right)$ denotes the sigmoid activation function.

### 2.8 Model training and evaluation

To mitigate the effect of class imbalance, weighted binary cross-entropy loss was adopted during training (Equation 14):

$$L=-\left[w_{+}\text{ylog}\left(\hat{y}\right)+\left(1-y\right)\log\left(1-\hat{y}\right)\right]$$ (14)

where $y\in \left\{0,1\right\}$ denotes the ground-truth label, $\hat{y}$ denotes the predicted probability, and $w_{+}$ denotes the weight assigned to positive samples. To account for class imbalance, the positive-class weight was calculated exclusively from the original training data as Equation 15:

$$w_{+}=\frac{N_{\text{neg}}^{\text{train}}}{N_{\text{pos}}^{\text{train}}}$$ (15)

where $N_{\text{neg}}^{\text{train}}$ and $N_{\text{pos}}^{\text{train}}$ represent the numbers of negative and positive samples in the training set, respectively. Neither the validation set nor the independent test datasets were used to calculate $w_{+}$ , thereby avoiding information leakage from the evaluation datasets.

The model was trained with a batch size of 8 for 50 epochs using an initial learning rate of $1\times 10^{-4}$ and a weight decay coefficient of $1\times 10^{-4}$ . The dropout rate was set to 0.35 to reduce overfitting. Model optimization was performed using AdamW, and the learning rate was adjusted with a cosine annealing schedule:

$$\eta_{t}=\eta_{\min}+\frac{1}{2}\left(\eta_{\max}-\eta_{\min}\right)\left(1+\cos\frac{t\pi }{T}\right)$$ (16)

where $\eta_{t}$ denotes the learning rate at epoch t , and T is the total number of training epochs. Gradient clipping and dropout were employed to improve training stability and reduce overfitting.

The predictive performance of AIPFusion-Net was evaluated using Accuracy, Sensitivity, Specificity, Matthews correlation coefficient, and the area under the receiver operating characteristic curve (Sui et al., 2025; Sui et al., 2024; Li et al., 2024; Wei et al., 2017; Li et al., 2025; Zhang et al., 2019; Song et al., 2018; Sui et al., 2023). These metrics are defined as Equations 17–20.

$$Acc=\frac{TP+TN}{TP+TN+FP+FN}$$ (17)

$$Sn=\frac{TP}{TP+FN}$$ (18)

$$Sp=\frac{TN}{TN+FP}$$ (19)

$$MCC=\frac{TP\times TN-FP\times FN}{\sqrt{\left(TP+FP\right)\left(TP+FN\right)\left(TN+FP\right)\left(TN+FN\right)}}$$ (20)

where TP , TN , FP , and FN represent true positives, true negatives, false positives, and false negatives, respectively. Accuracy measures the overall proportion of correctly classified samples. Sensitivity reflects the ability of the model to correctly identify AIPs, whereas Specificity measures the ability to correctly identify non-AIPs. MCC provides a balanced evaluation of classification performance, especially for imbalanced datasets. AUC measures the overall discriminative ability of the model across different classification thresholds; a higher AUC indicates better separation between AIPs and non-AIPs. These evaluation criteria were used to comprehensively assess the predictive capability and generalization performance of the proposed framework on the independent test sets. During model development, the original training dataset was randomly divided into a training subset (90%) and a validation subset (10%). The training subset was used for model parameter optimization, while the validation subset was used for hyperparameter selection and monitoring model performance during training. The independent test datasets (Ind-1049 and Ind-426) were not involved in model optimization and were exclusively used for the final evaluation of model generalization performance. To reduce the influence of random initialization and improve reproducibility, all experiments were independently repeated using three random seeds (42, 2024, and 3407), and the reported metrics were averaged across the three runs. To assess the generalization gap, nonparametric bootstrap analysis with 2,000 resamples was performed using AUC, with ΔAUC defined as AUC_test − AUC_training. Percentile-based 95% confidence intervals (CIs) were estimated from the bootstrap distribution, and differences were considered significant when the 95% CI excluded zero.For comparisons among local window configurations, paired bootstrap analysis with 2,000 resamples was used to compare the full-window setting with the best single- and dual-window settings on each independent test dataset. The same resampled samples were used for both configurations, and ΔAUC was defined as AUC_full-window − AUC_comparator.

## 3 Results

### 3.1 Performance comparison with existing methods

To comprehensively evaluate the effectiveness of AIPFusion-Net, we compared it with representative AIP prediction methods on two benchmark datasets, Ind-1049 and Ind-426. To ensure a fair and meaningful comparison, baseline methods were selected according to the comparability of experimental settings. Specifically, we followed the evaluation protocols adopted in recent AIP prediction studies (e.g., IF-AIP) and included methods evaluated on the same benchmark datasets using consistent evaluation metrics. The compared methods included AIPpred, PreAIP, AIEPred, iAIPs, IF-AIP, and AntiInflam where applicable. Although other AIP prediction methods, such as DAC-AIPs, DeepAIP, PepNet, and AIPs-SnTCN, have been reported, they were not included in the quantitative comparison because some were evaluated using different datasets or experimental settings, and some lacked publicly available source codes or sufficient reproducibility details for evaluation under the same experimental framework. Five evaluation metrics were used for performance assessment: Accuracy (ACC), Sensitivity (SN), Specificity (SP), Matthews correlation coefficient (MCC), and the area under the ROC curve (AUC).

On the Ind-1049 dataset (Figure 2), AIPFusion-Net achieved the best overall performance, with an average ACC of 84.6%, SN of 71.7%, SP of 89.3%, MCC of 0.654, and AUC of 0.884 across three independent runs using different random seeds (Supplementary Table S1). Compared with IF-AIP, the strongest competing method, AIPFusion-Net improved ACC by 4.6 percentage points, MCC by 0.075, and AUC by 0.011. Although its SN was slightly lower than that of AIPpred and its SP was slightly lower than that of AIEPred, AIPFusion-Net obtained the highest ACC, MCC, and AUC, indicating better overall predictive reliability and discriminative ability. The ROC curves in Figure 3 further confirm its superior performance on this dataset.On the Ind-426 dataset (Figure 4), AIPFusion-Net also showed the best comprehensive performance, achieving an average ACC of 82.3%, SN of 84.4%, SP of 76.9%, MCC of 0.673, and AUC of 0.882 across three independent runs (Supplementary Table S2). Compared with IF-AIP, AIPFusion-Net improved ACC by 4.6 percentage points, SN by 4.1 percentage points, SP by 2.7 percentage points, MCC by 0.145, and AUC by 0.011. Although AIEPred obtained the highest SP of 88.3%, its SN was only 52.3%, suggesting an imbalanced prediction tendency. In contrast, AIPFusion-Net achieved the highest ACC, SN, MCC, and AUC, demonstrating a more balanced and robust classification performance. This result is also supported by the ROC curves in Figure 5.

**FIGURE 2:**
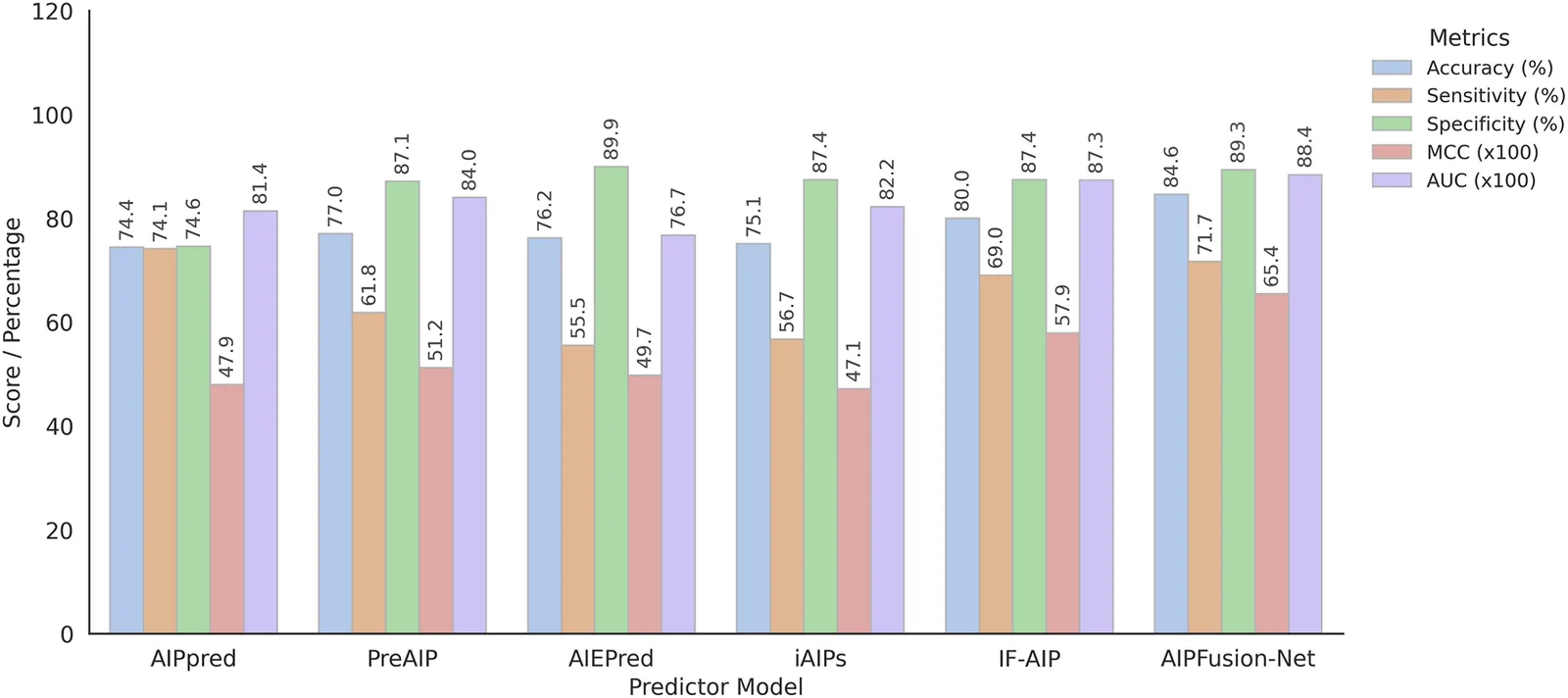
Performance comparison of AIPFusion-Net and state-of-the-art predictors on the Ind-1049 dataset.

**FIGURE 3:**
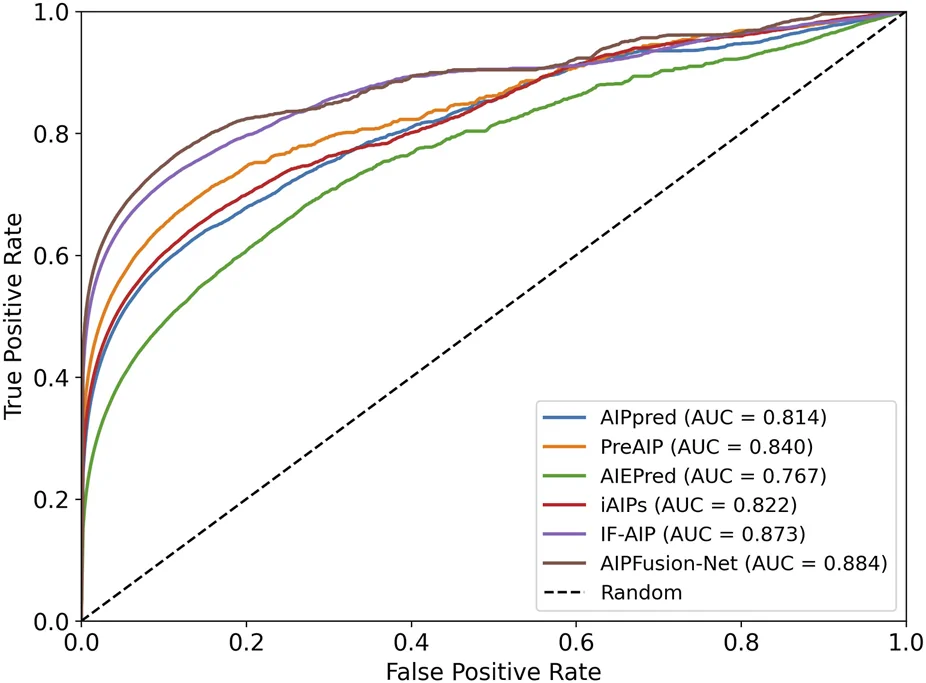
ROC curves of different predictors on the Ind-1049 dataset.

**FIGURE 4:**
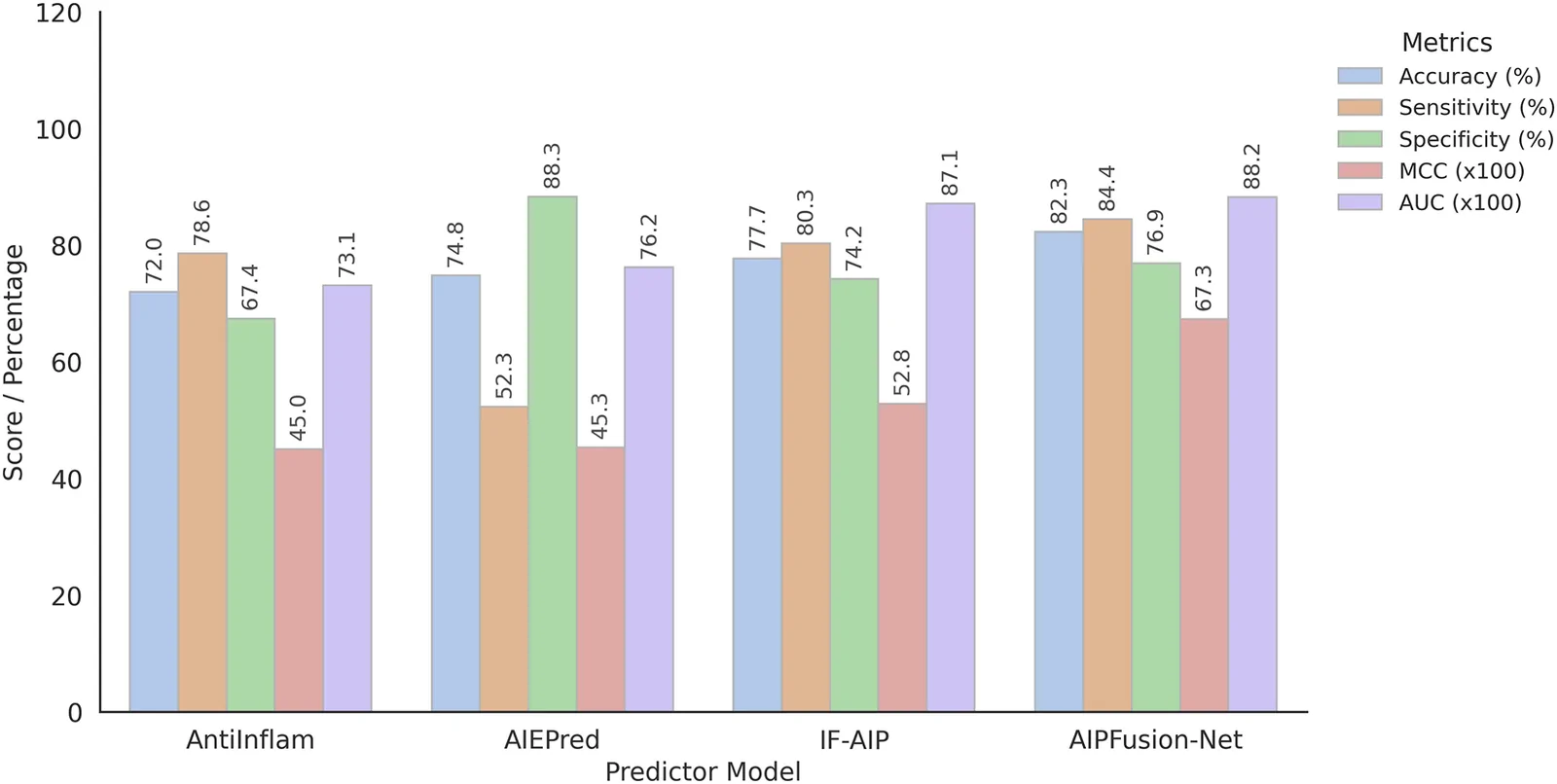
Performance comparison of AIPFusion-Net and state-of-the-art predictors on the Ind-426 dataset.

**FIGURE 5:**
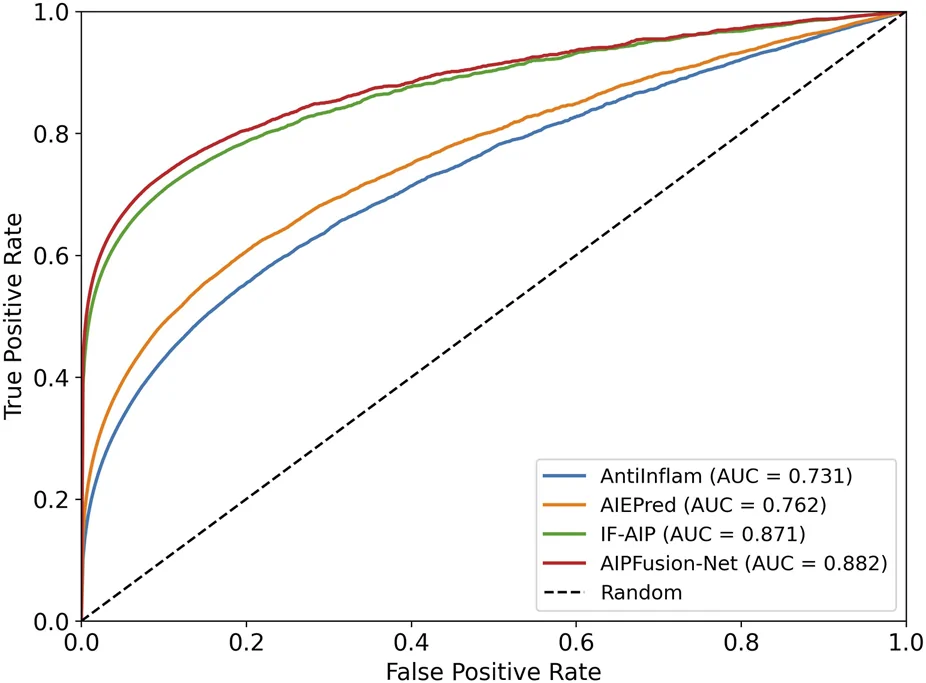
ROC curves of different predictors on the Ind-426 dataset.

Overall, AIPFusion-Net consistently achieved superior or highly competitive results across the two benchmark datasets. In particular, it obtained the highest ACC, MCC, and AUC on Ind-1049, and the highest ACC, SN, MCC, and AUC on Ind-426. These results demonstrate that AIPFusion-Net is a robust and reliable predictor for anti-inflammatory peptide identification.

### 3.2 Ablation analysis of feature combinations and interaction fusion strategy

To investigate the contribution of different feature sources and validate the effectiveness of the proposed interaction fusion strategy, we conducted comprehensive ablation experiments. Specifically, we evaluated models using handcrafted features, local features, global features, pairwise feature combinations, and the complete AIPFusion-Net. In addition, to determine whether the performance improvement was attributed to the proposed interaction fusion mechanism rather than simple feature integration, we constructed a fusion-concatenation variant by replacing the interaction fusion module with a conventional feature concatenation operation while keeping the same input representations and classifier architecture. The results of feature combination experiments are summarized in Tables 2 and 3, while the detailed comparison between interaction fusion and feature concatenation is provided in Supplementary Tables S3 and S4.

**TABLE 2:** Performance of different feature combinations on Ind-1049 dataset.

Feature combination	Accuracy (%)	Sensitivity (%)	Specificity (%)	MCC	AUC
Handcrafted only	69.8	52.6	77.1	0.303	0.712
Local only	76.9	61.4	83.5	0.462	0.793
Global only	80.4	67.8	85.8	0.561	0.844
Local + handcrafted	78.2	63.1	84.6	0.491	0.814
Global + handcrafted	81.3	68.9	86.4	0.583	0.855
Global + local	83.2	70.5	88.2	0.624	0.876
AIPFusion-Net	84.6	71.7	89.3	0.654	0.884

**TABLE 3:** Performance of different feature combinations on Ind-426 dataset.

Feature combination	Accuracy (%)	Sensitivity (%)	Specificity (%)	MCC	AUC
Handcrafted only	68.7	70.1	66.0	0.363	0.702
Local only	75.1	77.4	70.7	0.513	0.791
Global only	78.8	80.9	74.8	0.596	0.835
Local + handcrafted	76.4	78.6	72.2	0.547	0.805
Global + handcrafted	79.5	81.6	75.6	0.617	0.843
Global + local	81.1	83.0	76.5	0.648	0.873
AIPFusion-Net	82.3	84.4	76.9	0.673	0.882

On the Ind-1049 dataset (Table 2), the handcrafted-only model showed the lowest performance, with an Accuracy of 69.8%, MCC of 0.303, and AUC of 0.712. This suggests that handcrafted descriptors alone have limited discriminative capacity. In contrast, the local-only and global-only models achieved better results, with Accuracy values of 76.9% and 80.4%, respectively. The global-only model outperformed the local-only model across all major metrics, indicating that global sequence representations provide more informative features for this task. Among the pairwise combinations, the Global + Local model achieved the best performance, with an Accuracy of 83.2%, MCC of 0.624, and AUC of 0.876. Compared with the global-only model, this combination improved Accuracy by 2.8 percentage points and MCC by 0.063, suggesting that local contextual features can complement global representations. After further incorporating handcrafted features, the full AIPFusion-Net achieved the highest performance, with an Accuracy of 84.6%, Sensitivity of 71.7%, Specificity of 89.3%, MCC of 0.654, and AUC of 0.884. A similar trend was observed on the Ind-426 dataset (Table 3). The handcrafted-only model again produced the weakest results, with an Accuracy of 68.7%, MCC of 0.363, and AUC of 0.702. The local-only and global-only models improved the Accuracy to 75.1% and 78.8%, respectively, further confirming the importance of deep feature representations. The Global + Local combination achieved strong performance, with an Accuracy of 81.1%, MCC of 0.648, and AUC of 0.873. The full AIPFusion-Net further improved the results to 82.3% Accuracy, 84.4% Sensitivity, 76.9% Specificity, 0.673 MCC, and 0.882 AUC, outperforming all individual feature types and partial combinations.

To further assess the specific contribution of the interaction fusion strategy, we compared AIPFusion-Net with the fusion-concatenation variant. On the Ind-1049 dataset (Supplementary Table S3), the concatenation-based model achieved an Accuracy of 82.7%, Sensitivity of 67.9%, Specificity of 88.1%, MCC of 0.598, and AUC of 0.861, which were lower than those of AIPFusion-Net (Accuracy: 84.6%, Sensitivity: 71.7%, Specificity: 89.3%, MCC: 0.654, and AUC: 0.884). Compared with simple feature concatenation, the proposed interaction fusion strategy improved Accuracy by 1.9 percentage points, MCC by 0.056, and AUC by 0.023. Similarly, on the Ind-426 dataset (Supplementary Table S4), the concatenation-based model achieved an Accuracy of 80.1%, Sensitivity of 79.0%, Specificity of 75.2%, MCC of 0.612, and AUC of 0.857, whereas AIPFusion-Net achieved higher performance with an Accuracy of 82.3%, Sensitivity of 84.4%, Specificity of 76.9%, MCC of 0.673, and AUC of 0.882.

These results demonstrate that directly concatenating heterogeneous features cannot fully exploit the complementary relationships among different representations, whereas the proposed interaction fusion module enables more effective integration of global contextual information, local sequence patterns, and physicochemical properties.

Overall, the ablation results demonstrate that the superior performance of AIPFusion-Net is derived from both the complementary contributions of multiple feature sources and the proposed interaction fusion strategy. While handcrafted descriptors alone provide limited predictive ability, they introduce valuable physicochemical information when combined with deep representations. Moreover, compared with simple feature concatenation, the interaction fusion module can explicitly model the relationships among heterogeneous feature spaces, leading to more discriminative and robust representations for anti-inflammatory peptide identification.

### 3.3 Generalization and loss-function ablation analysis

To further evaluate the robustness of AIPFusion-Net, we assessed both its generalization performance and the contribution of class weighting. First, a nonparametric bootstrap analysis was performed to compare the AUC between the training dataset and the two independent test datasets (Supplementary Table S5). The training AUC was 0.915. On Ind-1049, the mean AUC across three independent runs was 0.884, corresponding to a ΔAUC of −0.031 (95% CI: −0.043 to −0.019, P < 0.001). On Ind-426, the mean AUC was 0.882, yielding a ΔAUC of −0.033 (95% CI: −0.054 to −0.015, P < 0.001). Although both independent test datasets showed statistically significant decreases in AUC compared with the training dataset, the absolute reductions were modest. Moreover, AIPFusion-Net maintained AUC values of approximately 0.88 on both independent datasets, demonstrating stable discriminative performance and good generalization to unseen peptide sequences.

We further evaluated the effect of class weighting by comparing AIPFusion-Net trained with weighted binary cross-entropy (WBCE) with the same model trained using standard binary cross-entropy (BCE), while keeping all other experimental settings unchanged (Supplementary Table S6). On Ind-1049, WBCE increased sensitivity from 65.8% to 71.7%, MCC from 0.621 to 0.654, and AUC from 0.875 to 0.884, while ACC increased from 83.9% to 84.6%. On Ind-426, sensitivity increased from 79.0% to 84.4%, MCC from 0.637 to 0.673, and AUC from 0.876 to 0.882, with ACC increasing from 81.7% to 82.3%. Although specificity decreased with WBCE, the consistent improvements in sensitivity and MCC, together with modest increases in AUC, indicate that class weighting improved positive-class recognition and provided a more balanced predictive performance under class imbalance.

### 3.4 Analysis of local window design under the global–local fusion setting

To assess the effectiveness of the local window design in AIPFusion-Net, we compared seven local window configurations on the independent test sets, including single-window, dual-window, and full-window settings. Unlike the feature-combination analysis in Section 3.2, this experiment focused specifically on the effect of local window construction under the global–local fusion setting. The global branch was kept unchanged, and only the local window configurations were varied before being fused with the global representation.As shown in Tables 4 and 5, the full-window configuration consistently achieved the best overall performance, suggesting that different local regions provide complementary sequence information.

**TABLE 4:** Analysis of different local window designs on Ind-1049 dataset.

Local window design	Accuracy (%)	Sensitivity (%)	Specificity (%)	MCC	AUC
N-terminal only	73.8	57.6	80.7	0.393	0.747
C-terminal only	74.4	58.9	81.0	0.403	0.757
Cleavage-centered only	79.8	66.9	85.3	0.546	0.826
N-terminal + C-terminal	77.6	63.2	83.7	0.497	0.791
N-terminal + cleavage-centered	82.4	69.7	87.8	0.617	0.863
C-terminal + cleavage-centered	81.7	68.8	87.2	0.595	0.852
N-terminal + C-terminal + cleavage-centered	83.2	70.5	88.2	0.624	0.876

**TABLE 5:** Analysis of different local window designs on Ind-426 dataset.

Local window design	Accuracy (%)	Sensitivity (%)	Specificity (%)	MCC	AUC
N-terminal only	72.6	74.8	68.4	0.435	0.732
C-terminal only	73.1	75.2	69.1	0.445	0.748
Cleavage-centered only	78.6	80.7	74.6	0.584	0.829
N-terminal + C-terminal	76.5	78.4	72.9	0.523	0.785
N-terminal + cleavage-centered	80.5	82.4	76.8	0.631	0.856
C-terminal + cleavage-centered	79.9	81.9	76.0	0.614	0.841
N-terminal + C-terminal + cleavage-centered	81.1	83.0	76.5	0.648	0.873

On the Ind-1049 dataset (), the cleavage-centered window performed best among the single-window settings, achieving an Accuracy of 79.8%, MCC of 0.546, and AUC of 0.826. It clearly outperformed the N-terminal-only and C-terminal-only settings across all metrics, indicating that cleavage-centered regions contain more informative local patterns. Among the dual-window settings, N-terminal + cleavage-centered achieved the strongest performance, with an Accuracy of 82.4%, MCC of 0.617, and AUC of 0.863. The full-window configuration further improved the performance to 83.2% Accuracy, 0.624 MCC, and 0.876 AUC. To determine whether these improvements in AUC were statistically significant, paired nonparametric bootstrap analyses with 2,000 resampling iterations were performed (Supplementary Table S7). Compared with the best single-window configuration, the full-window setting improved the AUC by 0.050 (95% CI: 0.034 to 0.066, P < 0.001). Compared with the best dual-window configuration, the improvement was smaller but remained statistically significant (ΔAUC = 0.013, 95% CI: 0.004 to 0.022, P = 0.004).

A similar pattern was observed on the Ind-426 dataset (Table 5). The cleavage-centered-only model again outperformed the terminal-only variants, achieving an Accuracy of 78.6%, MCC of 0.584, and AUC of 0.829. Among the dual-window settings, N-terminal + cleavage-centered obtained the best performance, with an Accuracy of 80.5%, MCC of 0.631, and AUC of 0.856. The full-window configuration achieved the highest overall performance, with an Accuracy of 81.1%, Sensitivity of 83.0%, Specificity of 76.5%, MCC of 0.648, and AUC of 0.873. Paired bootstrap analysis further showed that the full-window configuration significantly outperformed the best single-window setting, with a ΔAUC of 0.044 (95% CI: 0.020 to 0.068, P < 0.001). The improvement over the best dual-window configuration was also statistically significant, although the magnitude of the difference was smaller (ΔAUC = 0.017, 95% CI: 0.003 to 0.031, P = 0.018).

Overall, these results indicate that cleavage-centered subsequences provide the most discriminative local information among the individual windows. However, no single local region fully captures the sequence information required for optimal prediction. Integrating the N-terminal, C-terminal, and cleavage-centered regions resulted in statistically significant improvements in AUC over the best single-window and dual-window configurations on both independent test datasets. These findings support the complementary contribution of different local sequence regions and demonstrate that the full-window design provides a more comprehensive local representation for AIP prediction.

### 3.5 Hyperparameter sensitivity analysis

To evaluate the robustness of AIPFusion-Net, we conducted sensitivity analyses on three key hyperparameters: local window size, fusion hidden dimension, and dropout rate. These parameters are related to local context modeling, feature fusion capacity, and regularization strength, respectively. All sensitivity analyses were performed on the validation subset derived from the training dataset, which was used for hyperparameter optimization. The independent test datasets (Ind-1049 and Ind-426) were not involved in hyperparameter selection and were reserved exclusively for the final model evaluation. In this section, we present the sensitivity analysis results using the Ind-1049 dataset as the reference dataset for visualization, because it contains a larger number of samples and provides a more comprehensive representation of model performance trends. The results are shown in Figures 6–8.

**FIGURE 6:**
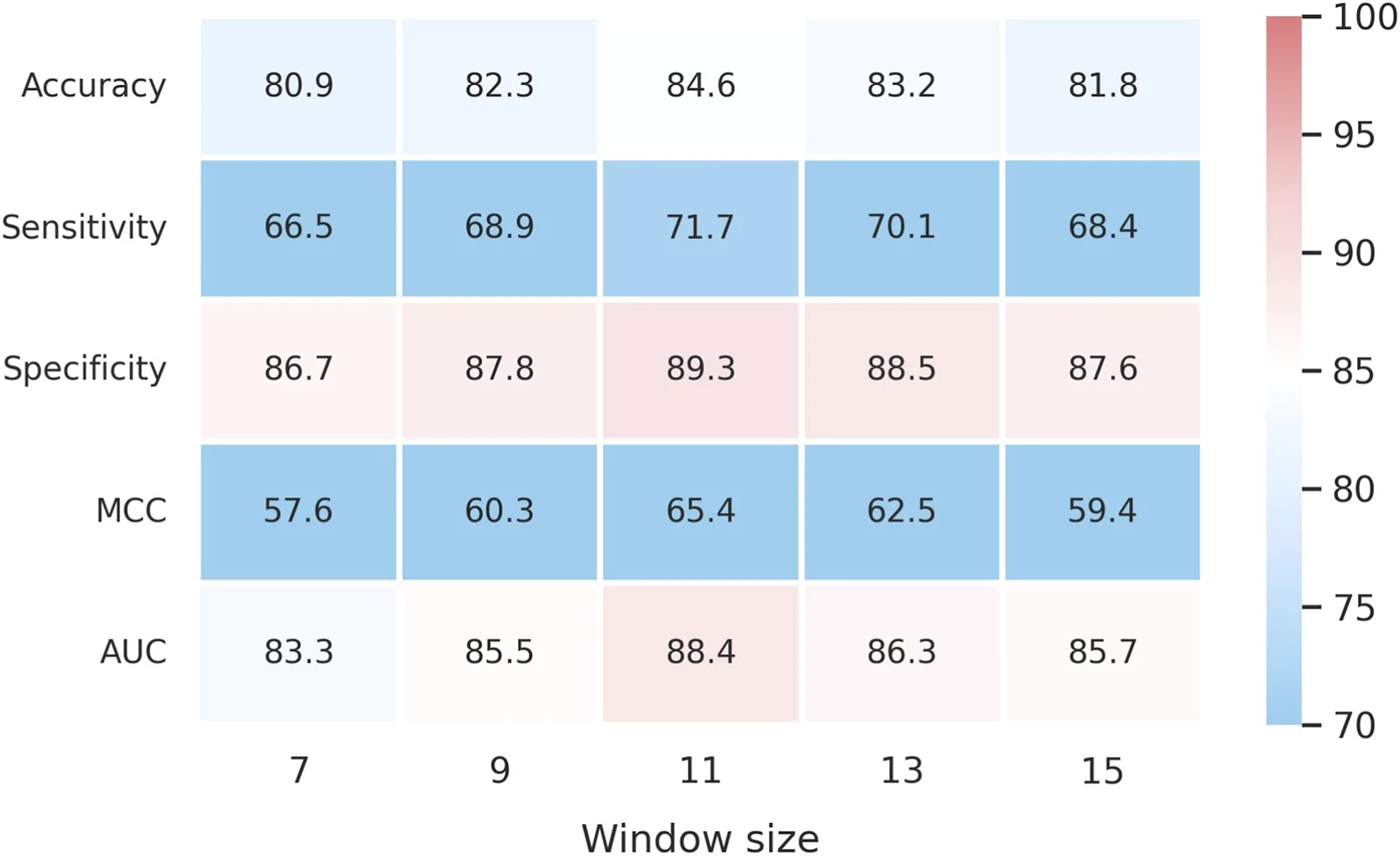
Sensitivity analysis of the local window size on the Ind-1049 dataset.

**FIGURE 7:**
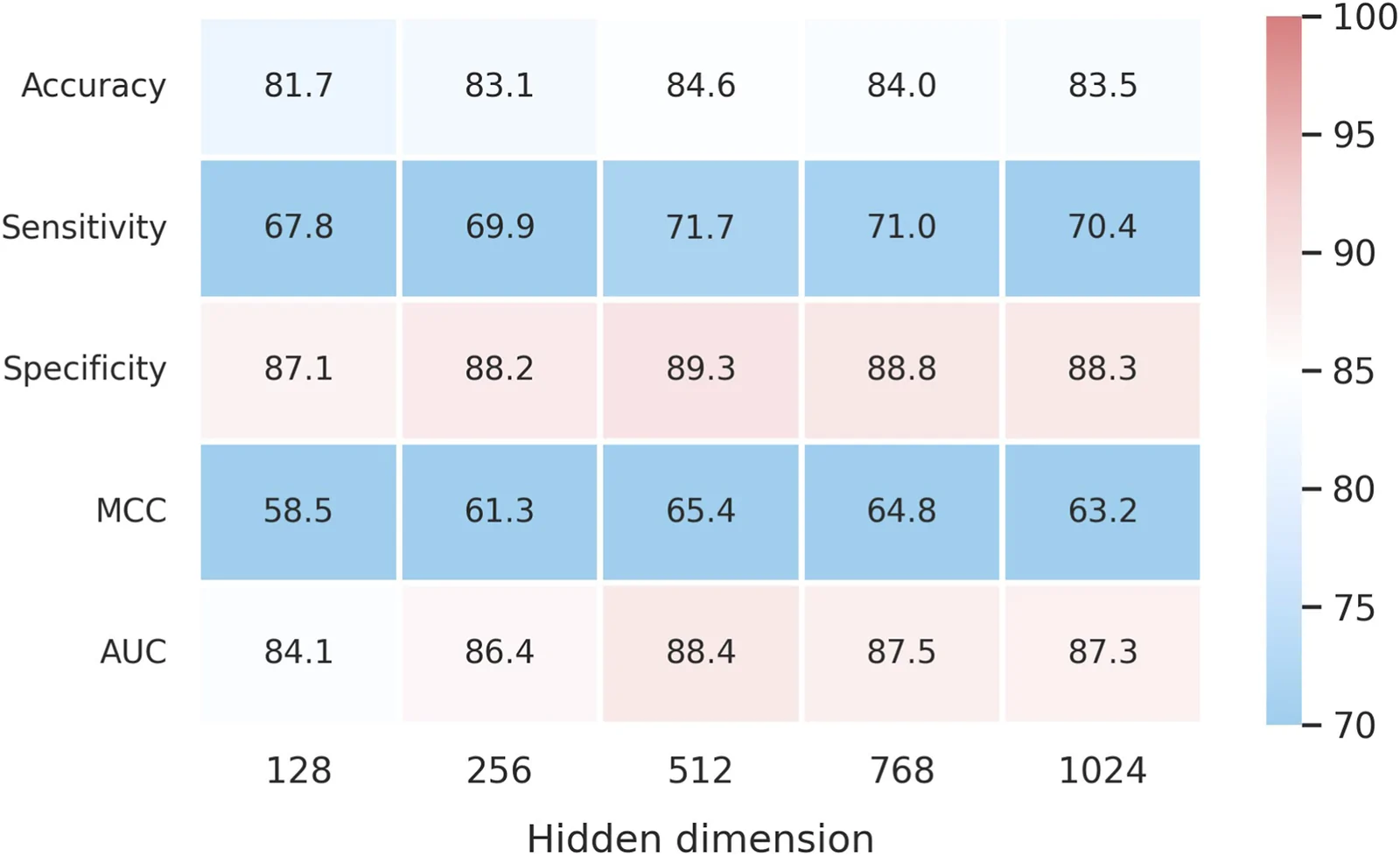
Sensitivity analysis of the fusion hidden dimension on the Ind-1049 dataset.

**FIGURE 8:**
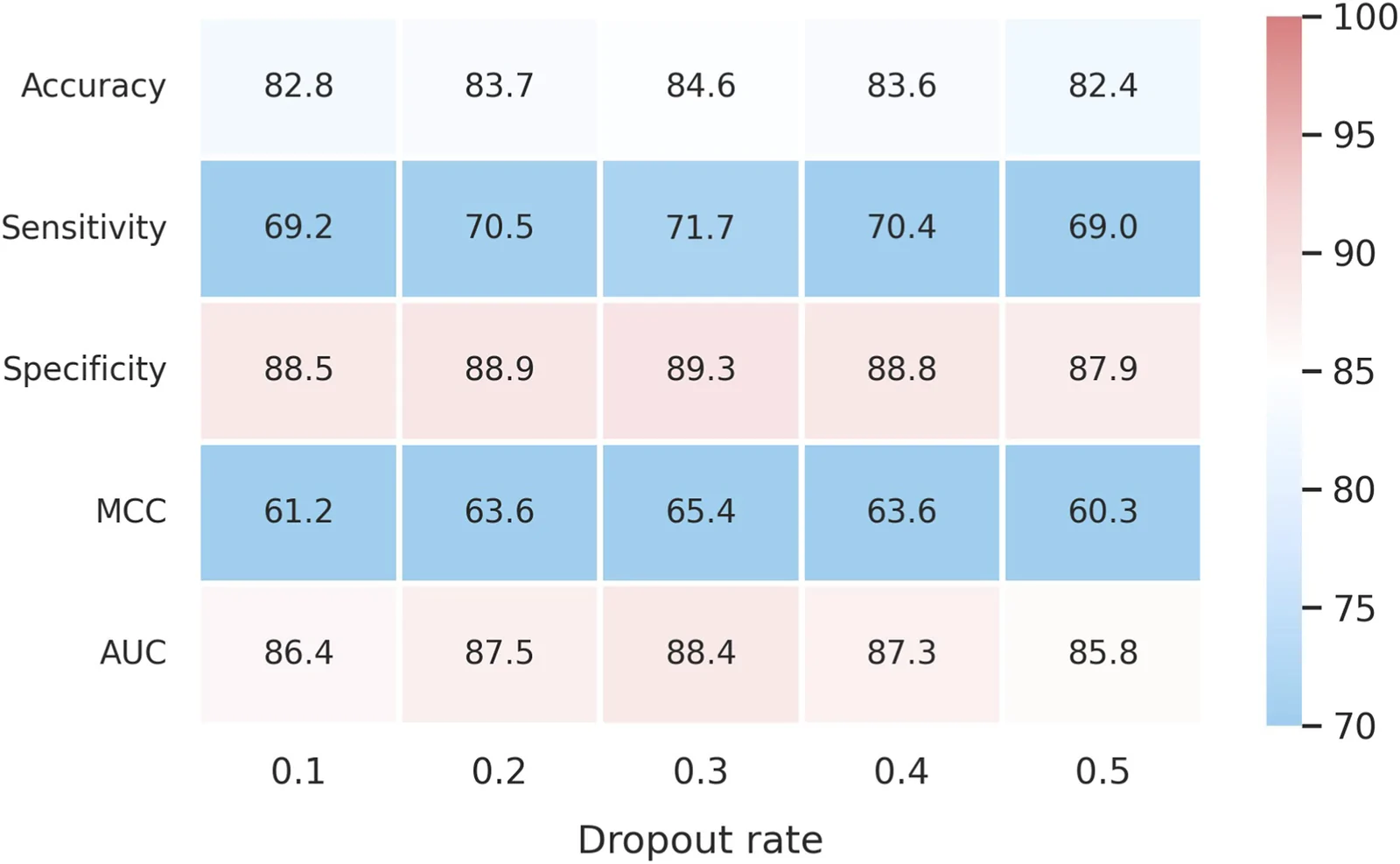
Sensitivity analysis of the dropout rate on the Ind-1049 dataset.

For the local window size (Figure 6), model performance increased as the window size expanded from 7 to 11, but gradually decreased when the window size was further enlarged. The best performance was achieved at a window size of 11, with an Accuracy of 84.6%, Sensitivity of 71.7%, Specificity of 89.3%, MCC of 0.654, and AUC of 0.884. This suggests that a moderate window size can capture sufficient local context while avoiding excessive noise from overly long regions.For the fusion hidden dimension (Figure 7), performance improved as the dimension increased from 128 to 512, reaching the optimal results at 512. Further increasing the dimension to 768 or 1024 did not improve performance and led to slight decreases. This indicates that an appropriate hidden dimension is sufficient for effective feature fusion, whereas an overly large dimension may introduce redundancy and increase model complexity.For the dropout rate (Figure 8), the best performance was obtained at 0.3, with the same optimal Accuracy, MCC, and AUC values of 84.6%, 0.654, and 0.884, respectively. Lower dropout rates may provide insufficient regularization, while higher dropout rates may suppress useful feature learning. The noticeable decline at a dropout rate of 0.5 suggests that excessive regularization can weaken the representation ability of the model.

Overall, the sensitivity analysis on the Ind-1049 dataset showed that AIPFusion-Net achieved optimal performance with a local window size of 11, a fusion hidden dimension of 512, and a dropout rate of 0.3. These findings suggest that the selected hyperparameter settings are appropriate for this dataset and that the model maintains relatively stable performance within a reasonable parameter range.

### 3.6 Feature visualization and model interpretation

To gain further insight into the effectiveness and interpretability of AIPFusion-Net, we performed feature visualization and attention-based interpretation analyses using the Ind-1049 dataset. PCA was applied to visualize the distributions of handcrafted features, global features, and the final fused features in a two-dimensional space, and residue-level attention maps were further used to reveal the sequence regions emphasized by the model during prediction.

As illustrated in Figure 9, feature separability improved progressively from handcrafted features to global features and finally to fused features. The handcrafted feature space showed substantial overlap between Non-AIP and AIP samples, reflecting the limited capacity of manually designed descriptors to distinguish the two classes. In comparison, the global feature space produced by the protein language model showed a clearer class boundary, indicating that pretrained embeddings captured richer and more discriminative semantic information. The fused feature space achieved the best visual separation, where the two classes formed more compact and distinct clusters with minimal overlap. This progressive improvement demonstrates that AIPFusion-Net can effectively combine heterogeneous feature representations and learn a more discriminative embedding space for AIP identification.

**FIGURE 9:**
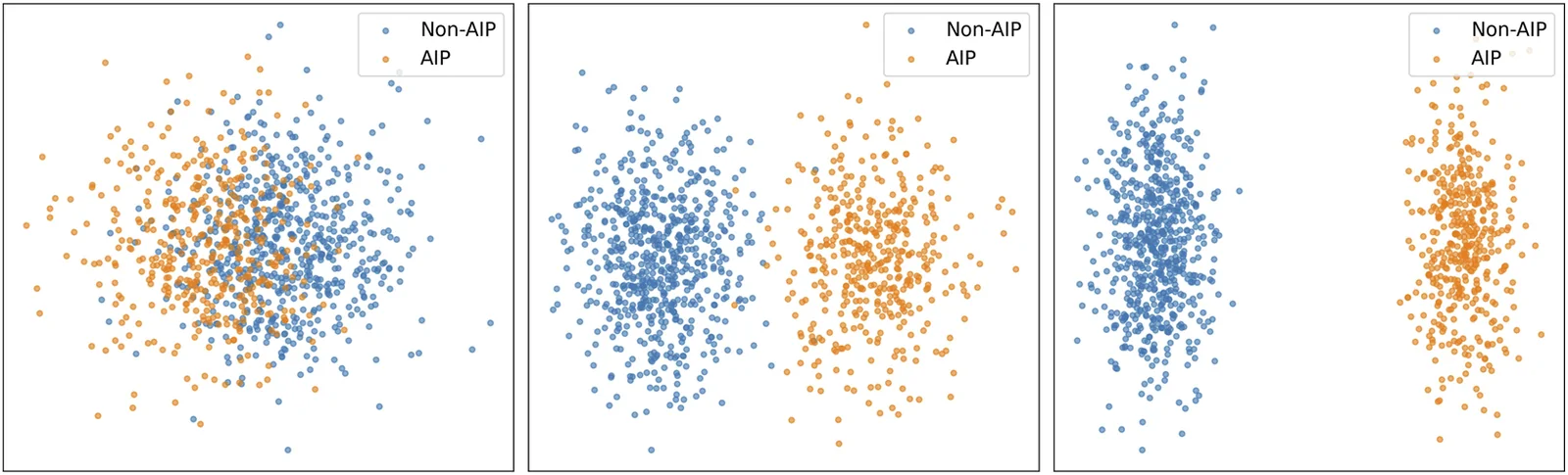
PCA visualization of handcrafted, global, and fused feature spaces for AIP classification.

To further interpret the model at the sequence level, we visualized the residue-level attention distributions for three representative peptide samples in the local branch, as shown in Figure 10. For S1, the model assigned relatively high attention to basic residues and several nearby hydrophobic or aromatic residues, indicating the importance of these regions in capturing functional patterns. For S2, the strongest attention was concentrated on basic residues such as lysine and arginine, suggesting that the model effectively identifies biologically relevant signals associated with peptide activity. In contrast, for S3, although basic residues also received elevated attention, the overall distribution was more dispersed across the sequence, reflecting weaker or less concentrated functional signals. These observations suggest that AIPFusion-Net is able to identify informative local regions and biologically meaningful residues during prediction.

**FIGURE 10:**
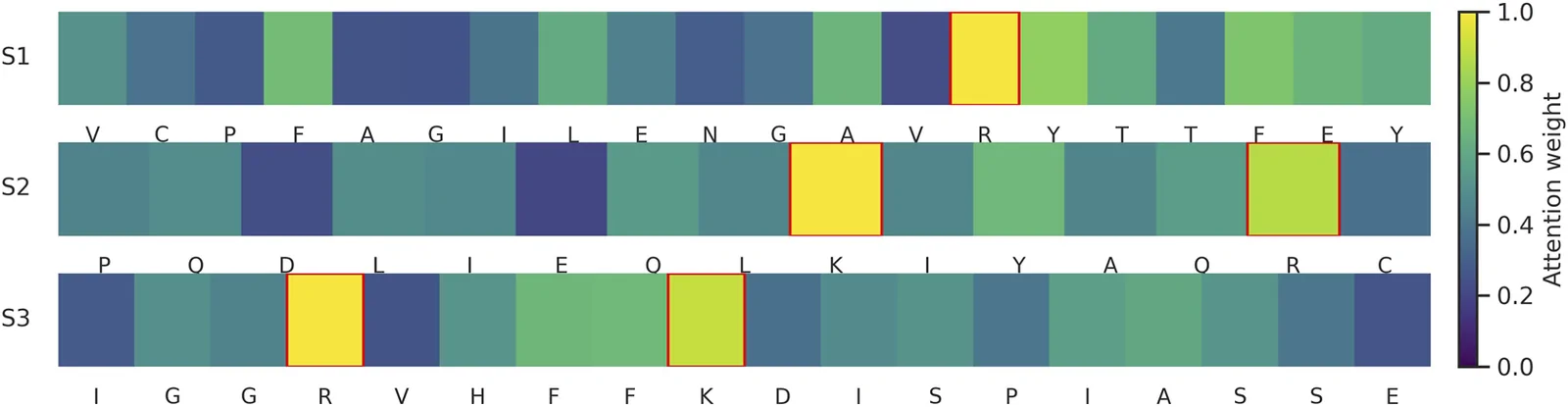
Residue-level attention maps of three representative peptide samples in the local branch.

Overall, the visualization results further support the effectiveness of AIPFusion-Net. The PCA analysis confirms that feature fusion improves representation quality, while the attention maps show that the model focuses on functionally relevant sequence regions in an interpretable manner.

## 4 Conclusion

In this study, we proposed AIPFusion-Net, an interaction-fusion framework for anti-inflammatory peptide prediction that integrates protein language model embeddings, local sequence patterns, and handcrafted descriptors. By combining global contextual information, biologically relevant local features, and sequence-derived physicochemical properties, the model learned more comprehensive and discriminative peptide representations. The interaction-fusion strategy further improved feature integration by explicitly capturing the relationships among heterogeneous representations. Experimental results on the Ind-1049 and Ind-426 datasets showed that AIPFusion-Net consistently outperformed existing methods in overall performance. In particular, it achieved the highest MCC and AUC values on both datasets, indicating better discriminative ability, stability, and generalization. Additional analyses also confirmed that global, local, and handcrafted features provide complementary information, and that their effective integration is important for optimal prediction performance. Although handcrafted descriptors alone were less discriminative than deep representations, they contributed useful physicochemical and compositional information when combined with global and local features, leading to more comprehensive peptide representations.

Overall, these results demonstrate that AIPFusion-Net is an effective and robust framework for AIP identification, with strong discriminative capability and generalization performance across different datasets. The proposed interaction-fusion mechanism highlights the importance of integrating heterogeneous biological features in a synergistic manner, providing a valuable reference for related peptide prediction tasks. In future work, incorporating structural information, such as predicted or experimentally determined 3D conformations, may further enhance the biological interpretability of the model. In addition, leveraging larger-scale pretrained protein language models and continually expanding high-quality, diverse benchmark datasets could improve model robustness and adaptability. Exploring transfer learning strategies and real-world peptide screening applications will also be important directions, potentially accelerating the discovery of novel anti-inflammatory peptides and facilitating their translation into practical biomedical use.

## Data Availability

The source code and datasets supporting the findings of this study are publicly available at: https://github.com/ChenYH7693/AIPFusion-Net.
