# Supplementary material for: AIPFusion-Net: an interaction-fusion framework for anti-inflammatory peptide prediction using protein language model embeddings, local sequence patterns, and handcrafted descriptors
Source: Front Mol Biosci. 2026 Oct 5;13:1952395. doi: 10.3389/fmolb.2026.1952395 (PMC13677489; doi:10.3389/fmolb.2026.1952395)
Supplement: Supplementary file 1 [file Table1.docx]

**Table S1.** Performance comparison of AIPFusion-Net across three independent runs with different random seeds on the Ind-1049 dataset.

| **Random seed** | **ACC (%)** | **SN (%)** | **SP (%)** | **MCC** | **AUC** |
| --- | --- | --- | --- | --- | --- |
| 42 | 84.1 | 70.8 | 88.9 | 0.646 | 0.879 |
| 2024 | 85.0 | 72.6 | 89.8 | 0.662 | 0.889 |
| 3407 | 84.7 | 71.8 | 89.3 | 0.655 | 0.884 |
| **Mean ± SD** | **84.6 ± 0.5** | **71.7 ± 0.9** | **89.3 ± 0.5** | **0.654 ± 0.008** | **0.884 ± 0.005** |

**Table S2.** Performance comparison of AIPFusion-Net across three independent runs with different random seeds on the Ind-426 dataset.

| **Random seed** | **ACC (%)** | **SN (%)** | **SP (%)** | **MCC** | **AUC** |
| --- | --- | --- | --- | --- | --- |
| 42 | 81.5 | 83.1 | 75.8 | 0.660 | 0.875 |
| 2024 | 83.0 | 85.8 | 78.0 | 0.685 | 0.889 |
| 3407 | 82.4 | 84.3 | 76.9 | 0.674 | 0.882 |
| **Mean ± SD** | **82.3 ± 0.8** | **84.4 ± 1.4** | **76.9 ± 1.1** | **0.673 ± 0.013** | **0.882 ± 0.007** |

**Table S3.**Comparison between interaction fusion and feature concatenation on Ind-1049.

| **Model** | **ACC (%)** | **SN (%)** | **SP (%)** | **MCC** | **AUC** |
| --- | --- | --- | --- | --- | --- |
| Concatenation-based fusion | 82.7 | 67.9 | 88.1 | 0.598 | 0.861 |
| AIPFusion-Net | **84.6** | **71.7** | **89.3** | **0.654** | **0.884** |

**Table S4.**Comparison between interaction fusion and feature concatenation on Ind-426.

| **Model** | **ACC (%)** | **SN (%)** | **SP (%)** | **MCC** | **AUC** |
| --- | --- | --- | --- | --- | --- |
| Concatenation-based fusion | 80.1 | 79.0 | 75.2 | 0.612 | 0.857 |
| AIPFusion-Net | **82.3** | **84.4** | **76.9** | **0.673** | **0.882** |

**Table S5.** Bootstrap-based statistical comparison of AUC between the training and independent test datasets.

| **Comparison** | **Training AUC** | **Test AUC** | **ΔAUC (Test − Training)** | **95% CI** | **P value** |
| --- | --- | --- | --- | --- | --- |
| Training vs Ind-1049 | 0.915 | 0.884 | −0.031 | −0.043 to −0.019 | <0.001 |
| Training vs Ind-426 | 0.915 | 0.882 | −0.033 | −0.054 to −0.015 | <0.001 |

**Table S6.** Ablation study comparing weighted binary cross-entropy and standard binary cross-entropy on the independent test datasets.

| **Dataset** | **Loss function** | **ACC (%)** | **SN (%)** | **SP (%)** | **MCC** | **AUC** |
| --- | --- | --- | --- | --- | --- | --- |
| Ind-1049 | BCE | 83.9 | 65.8 | **91.4** | 0.621 | 0.875 |
| Ind-1049 | WBCE | **84.6** | **71.7** | 89.3 | **0.654** | **0.884** |
| Ind-426 | BCE | 81.7 | 79.0 | **83.5** | 0.637 | 0.876 |
| Ind-426 | WBCE | **82.3** | **84.4** | 76.9 | **0.673** | **0.882** |

**Table S7**. Statistical comparison of AUC between the full-window configuration and the best single- and dual-window configurations.

| **Dataset** | **Comparator** | **Comparator AUC** | | **Full-window AUC** | **ΔAUC** | **95% CI** | **P value** |
| --- | --- | --- | --- | --- | --- | --- | --- |
| Ind-1049 | Best single-window | | 0.826 | 0.876 | 0.050 | 0.034–0.066 | <0.001 |
| Ind-1049 | Best dual-window | | 0.863 | 0.876 | 0.013 | 0.004–0.022 | 0.004 |
| Ind-426 | Best single-window | | 0.829 | 0.873 | 0.044 | 0.020–0.068 | <0.001 |
| Ind-426 | Best dual-window | | 0.856 | 0.873 | 0.017 | 0.003–0.031 | 0.018 |
